# Evaluation of fecal sample collection methods for feline gut microbiome profiling: fecal loop vs. litter box

**DOI:** 10.3389/fmicb.2024.1337917

**Published:** 2024-05-10

**Authors:** Xiaolei Ma, Emily Brinker, Christopher R. Lea, Diane Delmain, Erin D. Chamorro, Douglas R. Martin, Emily C. Graff, Xu Wang

**Affiliations:** ^1^School of Life Sciences and Technology, Tongji University, Shanghai, China; ^2^Department of Pathobiology, College of Veterinary Medicine, Auburn University, Auburn, AL, United States; ^3^Department of Comparative Pathobiology, Cummings School of Veterinary Medicine, Tufts University, North Grafton, MA, United States; ^4^Department of Clinical Sciences, College of Veterinary Medicine, Auburn University, Auburn, AL, United States; ^5^Scott-Ritchey Research Center, College of Veterinary Medicine, Auburn University, Auburn, AL, United States; ^6^Department of Anatomy, Physiology, and Pharmacology, College of Veterinary Medicine, Auburn University, Auburn, AL, United States; ^7^Center for Advanced Science, Innovation, and Commerce, Alabama Agricultural Experiment Station, Auburn, AL, United States; ^8^HudsonAlpha Institute for Biotechnology, Huntsville, AL, United States

**Keywords:** gut microbiota, fecal microbiome, stool sample collection, whole-genome shotgun metagenomic sequencing, microbial diversity

## Abstract

**Introduction:**

Microbial population structures within fecal samples are vital for disease screening, diagnosis, and gut microbiome research. The two primary methods for collecting feline fecal samples are: (1) using a fecal loop, which retrieves a rectal sample using a small, looped instrument, and (2) using the litter box, which collects stool directly from the litter. Each method has its own advantages and disadvantages and is suitable for different research objectives.

**Methods and results:**

Whole-genome shotgun metagenomic sequencing were performed on the gut microbiomes of fecal samples collected using these two methods from 10 adult cats housed in the same research facility. We evaluated the influence of collection methods on feline microbiome analysis, particularly their impact on DNA extraction, metagenomic sequencing yield, microbial composition, and diversity in subsequent gut microbiome analyses. Interestingly, fecal sample collection using a fecal loop resulted in a lower yield of microbial DNA compared to the litterbox method (*p* = 0.004). However, there were no significant differences between the two groups in the proportion of host contamination (*p* = 0.106), virus contamination (*p* = 0.232), relative taxonomy abundance of top five phyla (*Padj* > 0.638), or the number of microbial genes covered (*p* = 0.770). Furthermore, no significant differences were observed in alpha-diversity, beta-diversity, the number of taxa identified at each taxonomic level, and the relative abundance of taxonomic units.

**Discussion:**

These two sample collection methods do not affect microbial population structures within fecal samples and collecting fecal samples directly from the litterbox within 6 hours after defecation can be considered a reliable approach for microbiome research.

## Introduction

Understanding the feline microbiome is essential in veterinary medicine, informing the diagnosis and treatment of conditions such as gastrointestinal disorders, obesity, and immune-mediated diseases (Day, 2016; Suchodolski, 2016; Ma et al., 2022). Additionally, research on the feline microbiome offers insights into zoonotic disease transmission and the transfer of beneficial microorganisms between cats and their owners (Overgaauw et al., 2020; Bhat, 2021). Thus, investigating the feline microbiome is crucial for advancing veterinary medicine and enhancing our understanding of human-animal interactions. The method of collecting fecal samples is crucial for obtaining accurate microbial profiles in microbiome studies (Wang et al., 2018; Watson et al., 2019; Tang et al., 2020; Jones et al., 2021), providing insights into microbial population structures and their correlations with health or disease. The two most commonly used methods for collecting feline fecal samples are: (1) the fecal loop method, which involves using a small plastic instrument with a looped end to collect a sample of the cat’s stool from the rectum, and (2) the litter box approach, which involves collecting the cat’s stool directly from the litter box. For the latter approach, it is vital to collect the sample immediately after the animal defecates to minimize the risk of environmental contamination of the microbiome. The fecal loop method provides a precise and sanitary collection technique, which minimizes the risk of cross-contamination and exposure of anaerobes to oxygen. However, this approach is often invasive and potentially uncomfortable or painful for cats. It should only be performed by veterinarians or experienced personnel who can insert the loop into the rectum and gently scoop out a small amount of feces. Moreover, sedation may be required prior to fecal loop collection, which can increase the time and cost involved in the process, particularly when dealing with multiple cats. The litter box method involves regularly monitoring the litter box, and promptly collecting the fresh stool with a clean and sterile container or scoop when the cat defecates. This approach is a non-invasive and cost-effective method commonly used in large-scale population studies, involving sample collection by cat owners. However, there is a greater risk of introducing environmental contaminations, which may affect the accuracy and completeness of the microbial community representation in the sample (Hale et al., 2016; Tal et al., 2017; Tap et al., 2019). Collecting fecal samples directly from the litter box may limit the information available to the clinician and researcher regarding fecal consistency (Sherding and Johnson, 2006). The choice of method depends on factors such as the specific research goals, the need for precision and sanitation, the invasiveness and discomfort for the cat, and the potential for environmental contamination.

Researchers should be mindful of the potential limitations and take steps to minimize environmental contamination and ensure timely sample collection. In addition to the conditions of the fecal sample, the stability of the microbial community within fecal samples is a critical aspect of microbiome research. This is particularly important when considering the method of sample collection, as gut microbial profiles are often linked to health status and have the potential to indicate the development of metabolic diseases, gastrointestinal disorders, and even cancer (Fukuda and Ohno, 2014; Parekh et al., 2015; Sanz et al., 2015; Quigley, 2017; Gopalakrishnan et al., 2018; Gorkiewicz and Moschen, 2018; Dabke et al., 2019; Li et al., 2019; Akbar et al., 2022). Using a fecal loop may reduce environmental contamination, but it also poses the risk of contaminating the sample with cells from the host’s bowel wall or blood due to improper technique. Furthermore, it is important to note that using a fecal loop for sample collection may result in insufficient amounts of fecal material, which in turn could lead to an incomplete representation of the microbial community (Claassen-Weitz et al., 2020; Villette et al., 2021; Kennedy et al., 2023). Conversely, collecting fecal samples directly from the litter box may eliminate the risk of inadequate sample collection; however, it may also increase the likelihood of environmental contamination and the introduction of extraneous bacterial taxa into the samples. It is essential to note that fecal samples collected directly from litter boxes may not be collected promptly, which can lead to prolonged exposure to ambient conditions. Room temperature and oxygen levels are crucial environmental factors that influence the growth and survival of bacteria, potentially leading to changes in the composition of the gut microbiome. Research studies have shown that long-term storage at room temperature may alter the microbial diversity and community (Howell et al., 1996; Amir et al., 2017; Tal et al., 2017; Martin de Bustamante et al., 2021), leading to an inaccurate representation of the fecal microbiome. Oxygen levels significantly affect the growth and metabolic processes of both aerobic and anaerobic bacteria (Kennedy et al., 2023). This emphasizes consideration of environmental conditions when determining the optimal method for collecting cat fecal samples.

More than 10 previous studies have explored fecal collection and storage methods, examining variables such as temperature, storage duration at different temperatures, and the application of stabilizers like the OMNI-gene GUT kit, 95% ethanol, RNAlater, and other preservative solutions (Van der Waaij et al., 1994; Dominianni et al., 2014; Doukhanine et al., 2014; Flores et al., 2015; Loftfield et al., 2016; Song et al., 2016; Vogtmann et al., 2017; Wong et al., 2017; Burz et al., 2019; Conrads and Abdelbary, 2019; Papanicolas et al., 2019; Tap et al., 2019; Wu et al., 2019; Liang et al., 2020; Shalaby et al., 2020). While these studies have identified various methods to achieve stable microbial composition results, a universally accepted standard protocol has yet to emerge. This standard is crucial to the consistency, reliability, and comparability of results across studies. The majority of such studies concentrated on the methods of collecting and storing human fecal samples, while research on handling animal fecal samples is relatively limited. In the case of cats, the only prior study was our own research, which focused on the fecal loop collection method, specifically examining the use of lubricant versus no lubricant (Ma et al., 2022). This research is the first investigation into two fecal sample collection methods in cats, specifically examining the potential variances in gut microbiome composition resulting from the use of a fecal loop for collection compared to direct retrieval from a litter box. This research addresses a previously unexplored area by systematically comparing microbiome profiles derived from fecal samples collected via these two distinct methods. To assess the potential impact of various collection methods on the composition of the microbial community, we collected two sets of fecal samples from a group of cats housed in a controlled research environment. One set was collected using fecal loops, while the other was collected directly from the litter box. The collected samples underwent whole-genome shotgun metagenomic sequencing, followed by comprehensive analyses of microbial diversity, composition, and abundance at all taxonomic and gene levels. Our study aimed to provide valuable insights into the impact of different fecal collection methods and to contribute to the development of standardized protocols for collecting fecal samples in feline microbiome research.

## Materials and methods

### Study animals

The Auburn University Institutional Animal Care and Use Committee (IACUC) approved the study. Four intact female and six intact male cats, raised and maintained at the Scott-Ritchey Research Center, Auburn University College of Veterinary Medicine (Auburn, AL, USA), were enrolled in this study (Table 1). The age range of the 10 adult cats is 2.7–7.0 years old, with a mean age of 4.4 years. All cats are housed in USDA and AAALAC accredited facilities in indoor wards with heating and air conditioning that allow compliance with federally mandated climate control parameters including an ambient temperature of ~72 degrees Fahrenheit, ranging from 64 to 84 degrees, with humidity between 30 and 70%. Cats were allowed *ad libitum* access to food and water. They were fed a Hill’s Science Diet maintenance-formula dry food mixed with an equal amount of Friskies canned food. There was a rotation of the canned food protein sources (tuna, salmon, chicken, beef, and turkey) to increase enrichment. All cats were provided access to the same rotating protein source and there was no changes in diet throughout the study. The cats are born, raised, and housed in the colony and are maintained in these conditions throughout adulthood or until adoption. They were all cared for according to the principles outlined in the NIH Guide to the Care and Use of Laboratory Animals.

**Table 1:** Characteristics of study participants and fecal sample collection date/time.

Cat ID	Sex	Date of birth	Date of litterbox collection	Time of litterbox collection	Date of fecal loop collection	Time of fecal loop collection
9–1866	F	10/20/2015	10/4/2022	6:00	10/4/2022	13:00
944	F	3/17/2019	10/5/2022	14:00	10/10/2022	13:30
924	F	9/20/2018	9/27/2022	22:00	9/28/2022	8:20
960	F	1/25/2020	10/12/2022	12:00	10/13/2022	11:15
926	M	9/20/2018	9/28/2022	6:00	9/28/2022	12:00
936	M	1/21/2019	9/27/2022	18:00	9/28/2022	8:20
9–2033	M	5/5/2018	9/27/2022	6:00	9/28/2022	8:30
921	M	2/25/2018	10/4/2022	14:00	10/5/2022	14:00
9–2060	M	8/6/2018	9/28/2022	6:00	9/28/2022	12:00
9–1952	M	3/23/2017	9/28/2022	12:00	9/29/2022	15:00

F, Intact female; M, Intact male.

### Sample size determination

To perform a systematic comparison of microbiome profiles generated from fecal samples collected using these two methods, we collected two sets of fecal samples from these cats. One set was obtained using fecal loops, while the other was collected directly from the litter box.

In total, 20 fecal samples were collected from 10 cats. Our previous work has discovered that that more than 90% of microbial genes and species are covered in a feline microbiome study when the sample size reaches eight (Ma et al., 2022). In this study, we performed rarefaction analyses on the 20 samples in this study at both the gene level (Supplementary Figure S1A) and the species levels (Supplementary Figure S1B), through random subsampling from 20 samples multiple times and plotting the average gene and species richness against different numbers of included samples using a customized R script (Supplementary Data S1).

### Fecal sample collection and storage

Each cat was given 24 h to acclimate to a single housing environment. Afterward, each cat was provided with a fresh litter box and monitored every 2–6 h. After the cat defecated, the sample was immediately collected in a sterile 1.5 mL Eppendorf tube and stored at −80°C. The following morning, after collecting the fecal sample from the litterbox, the cat was sedated with intramuscular administration of medetomidine, ketamine, and butorphanol. A plastic fecal loop (Catalog number 7500, Covetrus, Dublin, OH, USA) was inserted into the rectum and descending colon to collect the fecal sample. The fecal loop was coated with mineral oil (Equate, Bentonville, AR, USA) as a lubricant, as described in our previous study (Ma et al., 2022). The samples were collected using 1.5 mL sterile Eppendorf tubes (Eppendorf, Hamburg, Germany) and immediately stored at −80°C (CryoCube F570, Eppendorf North America, Enfield, CT, USA) until analysis.

### Whole-genome shotgun metagenomic sequencing

The Qiagen Allprep PowerFecal DNA/RNA kit (Qiagen, Redwood City, CA, USA) was used for microbial DNA extraction. For each cat, the weight of fecal specimens was measured (Table 2) before being placed into a Microbial Lysis Tube for homogenization using a PowerLyzer24 instrument (Qiagen, Redwood City, CA, USA). DNA extraction procedures were conducted for all fecal samples in the same batch to minimize technical variability. The DNA concentrations were measured using a Qubit 3.0 Fluorometer (Thermo Fisher Scientific, Waltham, MA, USA), and the A260/A280 absorption ratios were determined with a NanoDrop One C Microvolume Spectrophotometer (Thermo Fisher Scientific, Waltham, MA, USA). 500 ng of DNA from each sample was fragmented into 500-bp fragments using an M220 Focused-ultrasonicator (Covaris, Woburn, MA, USA). The WGS metagenomic libraries were prepared using the NEBNext Ultra II DNA Library Prep Kit for Illumina (New England BioLabs, Ipswich, MA, USA). TapeStation 4,200 (Agilent Technologies, Santa Clara, CA, USA) was utilized to evaluate the library size distributions. Subsequently, the final libraries were quantified using qPCR before being sequenced on an Illumina NovaSeq6000 sequencing platform in 150-bp paired-end mode by Novogene Corporation Inc. in Sacramento, CA, USA.

**Table 2:** Amount of fecal material collected and DNA yield from fecal samples.

Cat ID	Group	Weight of feces collected (mg)	DNA yield (μg)	Group	Weight of feces collected (mg)	DNA yield (μg)
9–1866	LB	205	238	FL	112	181
944	LB	208	165	FL	165	113
924	LB	212	121	FL	192	134
960	LB	201	156	FL	190	39.4
926	LB	198	138	FL	160	89.2
936	LB	215	202	FL	100	179
9–2033	LB	219	150	FL	201	79.4
921	LB	202	193	FL	165	65.4
9–2060	LB	216	290	FL	212	226
9–1952	LB	212	286	FL	176	144

LB, fecal collection by picking from litterbox; FL, fecal collection by using fecal loop.

### Bioinformatic processing of metagenomic data

A total of 1.02 billion raw metagenomic reads, or 153 Gigabases (Gbp) of sequences, were generated from the 20 metagenomes (Table 3). The sequencing depth of coverage was 9.59 ± 2.04 per sample. Trimmomatic (version 0.36) (Bolger et al., 2014) was utilized to remove adapter sequences and low-quality bases. Host and viral sequences were eliminated by aligning the high-quality reads to the feline reference genome Felis_catus_9.0 (Buckley et al., 2020) and the viral genome downloaded from National Center for Biotechnology Information (NCBI) using Burrows-Wheeler Aligner (BWA) (v0.7.17-r1188) (Li and Durbin, 2009). The virus reference consists of 5,540 high-quality complete viral genomes curated by NCBI, with a total genome length of 166.4 megabases (Mb). The remaining microbial reads were extracted using SAMtools (version 1.17) (Li et al., 2009) and aligned to the feline gut microbiome reference contigs assembled from 16 Illumina short-read metagenomics data (GCA_022675345.1; short-read reference assembly) (Ma et al., 2022). To investigate whether different microbiome references will affect our analysis and conclusion, we also aligned metagenomic reads to the feline gut microbiome contigs assembled from Pacific Biosciences HiFi long-read using *N* = 8 fecal samples (accession number: PRJNA1062788; long-read reference assembly). The read mapping percentages against both short-read and long-read assemblies are summarized in Table 3.

**Table 3:** Whole-genome shotgun metagenomic sequencing yield, quality control, and alignment statistics.

Cat ID	Group	Total number of reads	% adapters & low-quality reads	% host sequences	% read alignment (reference 1)	% read alignment (reference 2)
9–1866	LB	42,054,216	2.23%	0.49%	96.82%	89.25%
944	LB	48,132,278	1.24%	0.08%	98.01%	92.79%
924	LB	46,459,964	0.72%	0.14%	97.81%	90.50%
960	LB	49,593,768	1.00%	0.30%	96.09%	87.73%
926	LB	55,893,016	0.95%	0.11%	98.41%	93.59%
936	LB	23,861,570	0.69%	2.48%	96.87%	86.83%
9–2033	LB	30,247,308	0.62%	2.49%	96.88%	86.84%
921	LB	47,944,568	0.75%	0.07%	97.84%	92.86%
9–2060	LB	68,800,896	0.63%	0.09%	98.41%	92.18%
9–1952	LB	49,365,474	0.61%	0.07%	98.52%	91.07%
9–1866	FL	42,659,160	0.75%	0.13%	93.65%	84.40%
944	FL	59,450,856	0.55%	0.66%	97.64%	88.93%
924	FL	58,907,188	0.53%	6.65%	97.83%	88.40%
960	FL	52,903,612	0.53%	3.48%	97.47%	90.17%
926	FL	61,076,576	0.62%	1.10%	97.90%	91.02%
936	FL	52,452,186	0.51%	1.31%	98.14%	87.30%
9–2033	FL	59,764,500	0.70%	38.98%	96.42%	85.48%
921	FL	53,357,488	0.49%	0.08%	97.09%	91.40%
9–2060	FL	63,409,540	0.51%	0.27%	98.41%	93.33%
9–1952	FL	56,489,430	0.51%	0.24%	98.28%	91.47%

LB, fecal collection by picking from litterbox; FL, fecal collection using fecal loop.

Reference 1: feline gut reference contigs from Illumina short-read metagenomic assembly (GCA_022675345.1).

Reference 2: feline gut reference contigs from Pacific Biosciences long-read metagenomic assembly (PRJNA1062788).

### Taxonomy assignment and quantification of taxonomy abundance

Taxonomy assignments were performed on reference contigs (Loftfield et al., 2016) against the NCBI-NR database using Kaiju (v1.7.3) (Menzel et al., 2016) to determine taxonomy annotations at the phylum, class, order, family, genus, and species levels. More than 90% of the reference contigs were annotated with the NCBI (National Center for Biotechnology Information) taxonomy ID. Based on the BWA alignments, read counts were obtained using BEDTools (version 2.30.0) (Doukhanine et al., 2014) with the command ‘bedtools coverage-f 0.9 -a region.bed -b reads.bam -counts’ (Quinlan and Hall, 2010). The taxonomy counts table was generated by aggregating the read counts of all contigs with the same taxonomy annotation using a custom Perl script. The taxonomy counts were then normalized by the total number of mapped reads in a sample to quantify the relative abundance of each taxonomic unit.

### Microbial diversity analyses

Alpha- and beta-diversity analyses were conducted on the microbial profiles at all taxonomic levels using the R package vegan (version 2.6–4) (Oksanen et al., 2013). The alpha diversity was assessed using the Shannon index (Shannon, 1997). The beta diversity was calculated based on the Bray-Curtis distance (Bray and Curtis, 1957) and visualized in the PCoA (Principal Coordinates Analysis) plot format. A permutational multivariate analysis of variance (PERMANOVA) test (Anderson, 2014) was performed to assess the centroids and dispersion of the LB (litter box) and FL (fecal loop) groups, based on the dissimilarity matrix.

### Microbial gene abundance analysis

Microbial gene predictions were performed on reference metagenomic contigs using MetaGeneMark (v3.38) (Zhu et al., 2010). The redundant genes were identified and combined using CD-HIT-est (v4.7) (Li and Godzik, 2006; Fu et al., 2012) with the criterion of global sequence identity exceeding 95%. To determine the gene abundance, per-gene read counts were extracted using “BEDtools coverage,” and gene abundance was normalized by RPKM (Reads Per Kilobase gene model per Million reads).

### Statistical analysis

The comparison of DNA yield, levels of host and viral contaminations, number of taxonomic units and microbial genes, alpha diversities, and relative abundance of each taxon between the LB and FL groups was conducted using the Wilcoxon signed-rank test (Bauer, 1972; Hollander et al., 2013) in the R software (R Core Team, 2013). For the multiple comparisons of the microbial profiles, we utilized the R package qvalue (Storey et al., 2015) to determine the false discovery rate. When the *p*-value was less than 0.05 or the *q*-value was less than 0.1, the null hypothesis was rejected. In addition to the pairwise nonparametric test, we also performed differential abundance testing using Analysis of Compositions of Microbiomes with Bias Correction (ANCOM-BC), which was implemented in the R package ANCOMBC (Lin and Peddada, 2020). To determine the differences in the variance, Levene’s test of equality of variances (Brown and Forsythe, 1974; Carroll and Schneider, 1985) and the Brown–Forsythe test (Iachine et al., 2010) were performed. To estimate the correlation of taxonomy composition in fecal samples between the LB and FL groups, Spearman’s rank correlation tests were conducted on the average relative abundance of taxa between the LB and FL groups using the “cor.test()” function from the stats R package (Table 4).

**Table 4:** Correlation of taxonomic abundance at phylum, class, order, family, genus, and species level between fecal loop (FL) and litter box (LB) groups.

Taxonomy level	% of FL taxa identified in LB (short-read assembly)	FL-LB abundance correlation (short-read assembly)	% of FL taxa identified in LB (long-read assembly)	FL-LB abundance correlation (long-read assembly)
Phylum	95.83%	0.9573	100.0%	0.9912
Class	97.80%	0.9804	100.0%	0.9915
Order	98.97%	0.9789	100.0%	0.9914
Family	96.97%	0.9698	100.0%	0.9912
Genus	94.07%	0.9373	99.21%	0.9876
Species	89.83%	0.8974	99.65%	0.9794

## Results

### Fecal sample collection using a fecal loop resulted in a lower DNA extraction yield compared to the litterbox method

**Figure 1:**
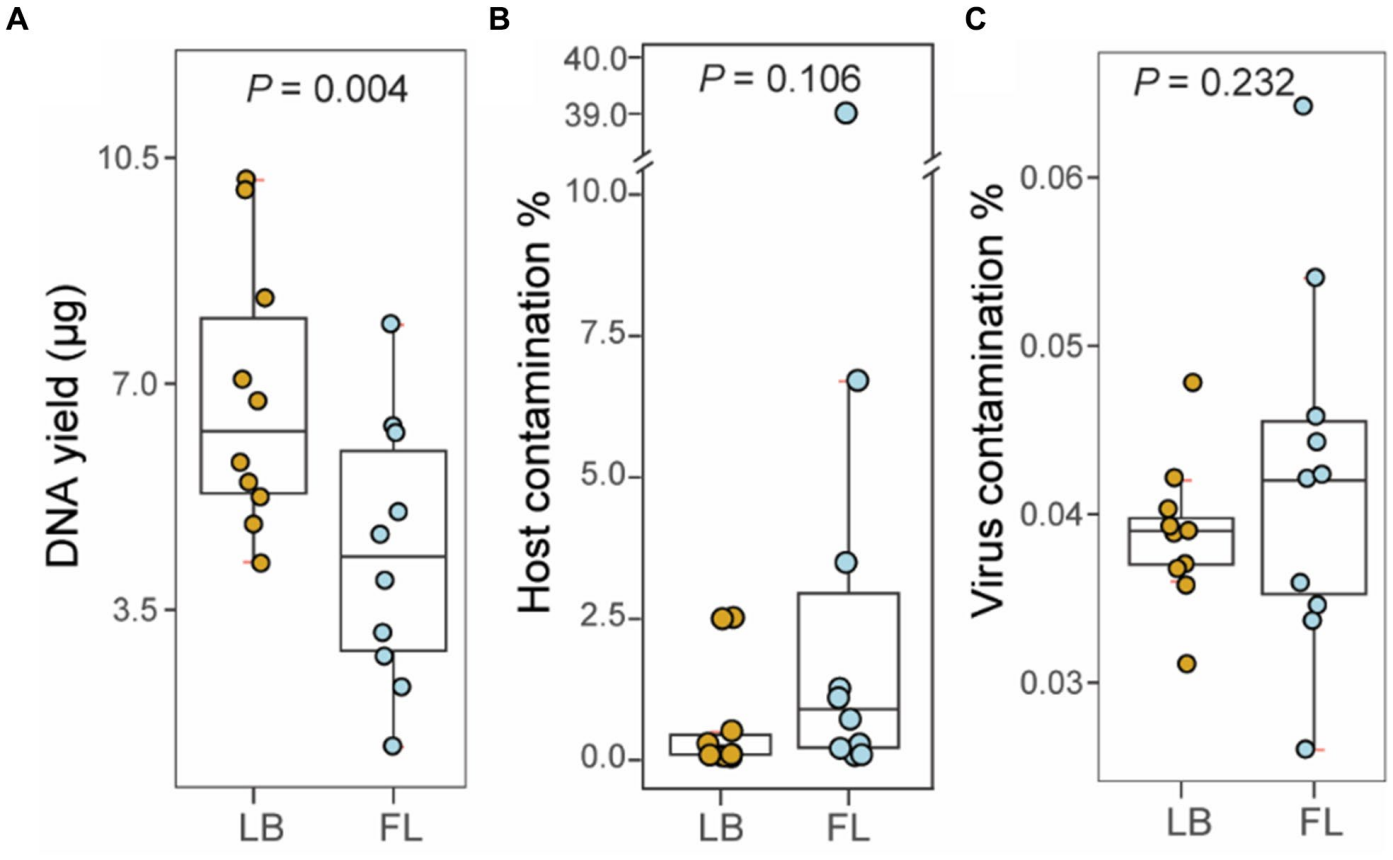
Metagenomic sequencing statistics from fecal samples collected by fecal loop (FL) and litter box (LB) approaches. **(A)** Boxplot of DNA yield (μg) extracted from fecal specimens in LB (brown) and FL (blue) groups. **(B)** Boxplot of percentage of host contamination in LB (brown) and FL (blue) groups. **(C)** Boxplot of percentage of viral contamination in LB (brown) and FL (blue) groups.

The amount of fecal material per sample collected using a fecal loop (FL group) was significantly lower than that collected from the litter box approach (*p* = 0.002, Wilcoxon signed-rank test; Table 2). As a result, the DNA yield of the FL group (4.376 μg [2.905 μg – 5.848 μg, 95% CI]) was significantly lower than that of the LB group (6.787 μg [5.285 μg – 8.288 μg, 95% CI]) (*p* = 0.004, Wilcoxon signed-rank test; Figure 1A). This suggests that the fecal collection method using a fecal loop might result in a reduced amount of DNA for subsequent research.

### No significant difference was observed in the levels of contaminants in the WGS metagenomic sequencing data between the LB and FL groups

A total of 1.02 billion 150-bp reads (153.4 Gbp of sequences) were generated in total through whole-genome shotgun (WGS) metagenomic sequencing of 20 fecal DNA samples (51.1 million reads per sample; Table 3). On average, 0.76% of the adapter sequences and low-quality bases were trimmed and excluded from subsequent analysis. The level of feline sequence contamination was 8-fold higher in the FL group (5.290% [−3.306–13.886%, 95% CI]) than in the LB groups (0.631% [−0.073–1.337% 95%, CI]), but the difference did not reach statistical significance (*p* = 0.11, Wilcoxon signed-rank test; Figure 1B). The levels of viral contamination did not show a significant difference between the LB group (0.039% [0.036–0.042%, 95% CI]) and the FL group (0.042% [0.035–0.050%, 95% CI]) (*p* = 0.232, Wilcoxon signed-rank test; Figure 1C). However, there were higher variations in host and viral sequence contamination detected in FL samples, with marginal significance (*p* = 0.05, Levene’s test of homogeneity of variance). When the Brown–Forsythe test was used, homogeneity of variances between the two groups cannot be rejected (*p* = 0.25).

### No significant differences were found in the number of microbial taxa discovered in the fecal specimens from the LB and FL groups

**Figure 2:**
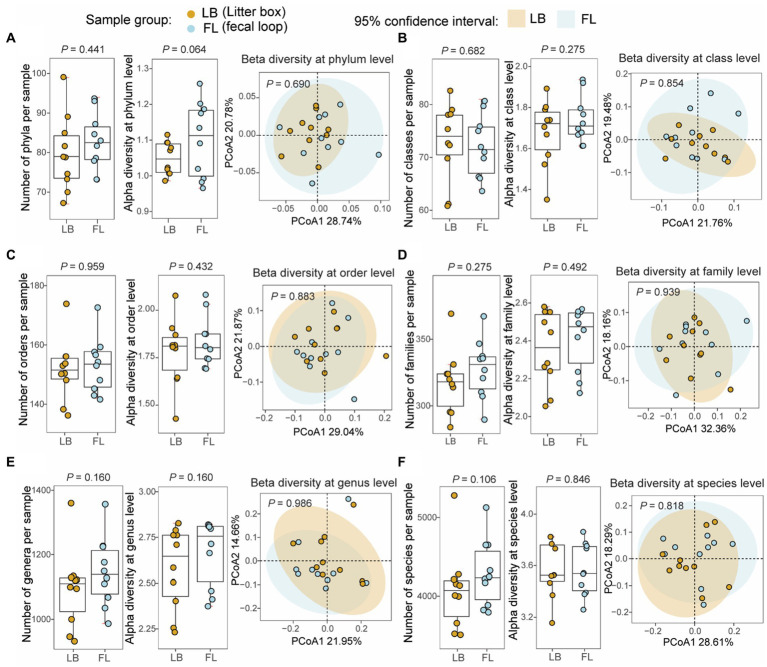
Microbial diversity analyses at different taxonomic levels from fecal samples collected by fecal loop (FL) and litter box (LB) approaches. Boxplots of non-redundant microbial taxa and alpha diversity (Shannon index) for each sample and principal coordinates analysis (PCoA) plot of beta diversity (Bray–Curtis dissimilarity) for microbial profiles from the LB (brown) and FL (blue) groups at **(A)** phylum, **(B)** class, **(C)** order, **(D)** family, **(E)** genus, and **(F)** species levels.

From the WGS metagenomic data, a total of 127 phyla, 93 classes, 196 orders, 435 families, 1,892 genera, and 8,467 species were identified in 20 samples based on the short-read reference assembly. No significant difference was observed in the number of microbial taxa between the LB (79.8 taxa [73.0–86.6, 95% CI]) and FL groups (82.7 [77.5–87.9, 95% CI]) at the phylum (*p* = 0.441, Wilcoxon signed-rank test), class (LB: 73.0 [67.7–78.3, 95% CI], FL: 72.1 [67.9–76.3, 95% CI], *p* = 0.682), order (LB: 151.5 [144.0–159.0, 95% CI], FL: 153.2 [146.5–159.9, 95% CI], *p* = 0.959), family (LB: 317.2 [300.2–334.2, 95% CI], FL: 327.7 [312.0–343.4, 95% CI], *p* = 0.275), genus (LB: 1093.9 [1007.2–1180.6, 95% CI], FL: 1146.2 [1069.5–1222.9, 95% CI], *p* = 0.160) and species levels (LB: 4074.3 [3709.4–4439.2, 95% CI], FL: 4288.0 [3985.3–4590.7, 95% CI], *p* = 0.106; Figure 2). The short-read assembly contains a large number of rare taxa, which greatly inflates the number of identified taxa due to ambiguity and false positives in taxonomic assignments. To address this issue, we aligned the metagenomic reads to an improved long-read feline gut microbiome assembly with enhanced metagenomic contig size and completeness. A total of 19 phyla, 35 classes, 63 orders, 104 families, 298 genera, and 936 species were identified using the long-read reference. When we repeated the analyses, we did not discover any significant differences in the number of microbial taxa between the groups either (Supplementary Figure S2).

### No significant variation in microbial diversities was observed at all taxonomic levels between the LB and FL groups

Alpha-diversity, as measured by the Shannon index, and beta-diversity, assessed using the Bray-Curtis distance, were determined for microbial profiles in both the LB and FL groups (Figure 2). For alpha-diversity, no significant differences were detected between the LB and FL groups at the phylum (LB: 1.05 [1.02–1.08, 95% CI], FL: 1.10 [1.03–1.18, 95% CI]; *p* = 0.064), class (LB: 1.68 [1.56–1.79, 95% CI], FL: 1.74 [1.66–1.82, 95% CI]; *p* = 0.275), order (LB: 1.78 [1.65–1.91, 95% CI], FL: 1.83 [1.74–1.93, 95% CI]; *p* = 0.432), family (LB: 2.36 [2.21–2.50, 95% CI], FL: 2.41 [2.29–2.53, 95% CI]; *p* = 0.492), genus (LB: 2.58 [2.42–2.74, 95% CI], FL: 2.67 [2.54–2.80, 95% CI]; *p* = 0.160), and species levels (LB: 3.57 [3.42–3.73, 95% CI], FL: 3.57 [3.42–3.71, 95% CI]; *p* = 0.846; Figure 2). When additional alpha diversity metrics were examined, we failed to discover any significant differences in Simpson diversity index, richness, or Chao1 index between FL and LB (*p* > 0.05). Similarly, no significant changes were detected in beta-diversity analysis either (*p* > 0.689 for all taxonomic levels, PERMANOVA test; Figure 2) using both Bray-Curtis and Jaccard distance measures. When we use the long-read assembled reference contigs as the mapping reference, the results remain consistent (see Supplementary Figure S2).

### Consistent relative taxonomic abundance in the microbiome quantified from fecal samples collected by LB and FL

**Figure 3:**
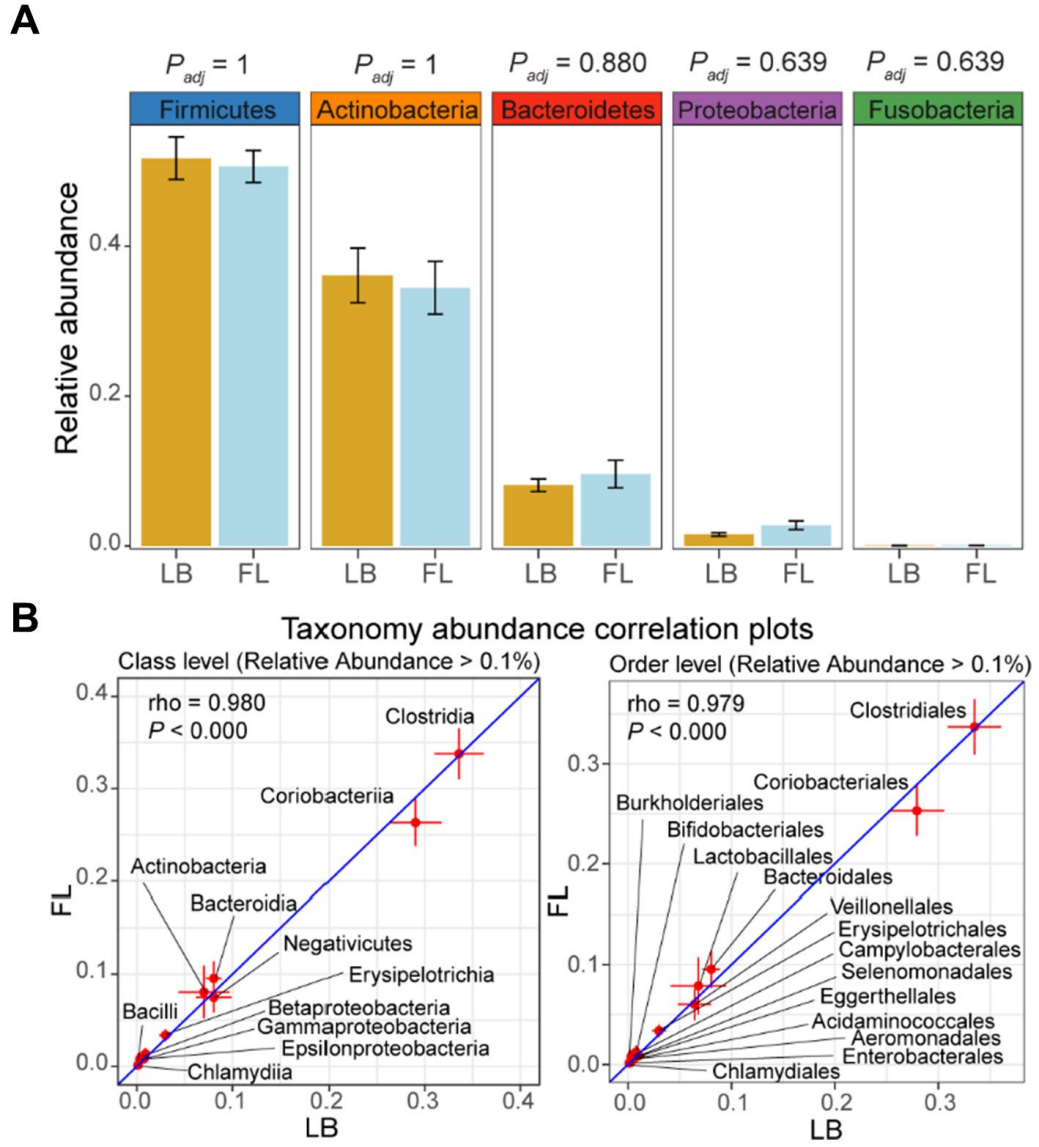
Relative abundance of major phyla and microbiome abundance (> 0.1%) correlation at class and order levels in the feline microbiome from samples collected by fecal loop (FL) and litter box (LB) approaches. **(A)** Boxplots of major phyla in LB (brown) and FL (blue) groups. **(B)** Correlation plots of microbes with high abundance (> 0.1%) at class and order levels. Each data point on the plot represents the averaged relative abundance of a particular taxon across samples within each group (FL on the *y*-axis and LB on the *x*-axis), and error bars indicate the standard error intervals around the mean for FL (vertical lines) and LB (horizontal lines) groups.

Through Wilcoxon signed-rank tests on all taxonomic categories at the phylum level in the LB and FL groups, no significant difference was detected in the relative abundance of the top five most abundant phyla: Firmicutes (LB: 48.6% [42.6–54.6%, 95% CI] vs. FL: 47.5% [43.1–51.9%, 95% CI]; *P_adj_* = 1), Actinobacteria (LB: 39.4% [30.8–47.9%, 95% CI] vs. FL: 37.7% [29.4–46.0%, 95% CI]; *P_adj_* = 1), Bacteroidetes (LB: 8.1% [6.1–10.1%, 95% CI] vs. FL: 9.6% [5.3–14.0%, 95% CI]; *P_adj_* = 0.880), Proteobacteria (LB: 0.9% [0.4–1.4%, 95% CI] vs. FL: 1.9% [0.6–3.3%, 95% CI]; *P_adj_* = 0.639), and Fusobacteria (LB: 0% [0–0%, 95% CI] vs. FL: 0% [0–0%, 95% CI]; *P_adj_* = 0.639; Figure 3A). Collectively, these five predominant phyla represented more than 97% of all phyla observed in both the LB and FL groups (97.6% [97.2–97.9%, 95% CI] vs. 97.5% [97.2–97.8%, 95% CI]; *p* = 1). When utilizing the long-read assembled feline gut microbiome contigs as the reference, the top five most abundant phyla remained consistent and maintained the same ranking order (Supplementary Figure S3A). Upon examining lower taxonomic units, there were no significant differences in the relative abundance between the LB and FL groups at the class, order, family, genus, or species levels (*P_adj_* > 0.909 for short-read assembly, and *P_adj_* > 0.379 for long-read assembly). Furthermore, in addition to pairwise nonparametric tests, we employed the ANCOM approach for detecting differential abundance as outlined in the Methods section. Our analysis did not reveal any taxa with a statistically significant difference in abundance between the LB and FL groups (FDR > 0.05; Supplementary Data S2), and 99.5% of the tested taxa exhibited an FDR = 1, suggesting remarkable concordance in microbial abundance between the two fecal sample collection methods.

### A strong correlation in taxonomic composition was observed among fecal samples in the LB and FL groups

When using the long-read assembled feline gut microbiome reference contigs, the LB and FL groups showed nearly perfect abundance correlation at phylum, class, order, and family levels, with Spearman’s rank-order correlation coefficients greater than 0.99 (Table 4 and Supplementary Figure S3B; *p* = 0.000; Spearman’s Rank-Order Correlation test). All taxa identified in the FL samples were also detected in the LB data (Table 4). At phylum, class, order, and family levels, results from short-read assembly demonstrated strong abundance correlations with lightly lower correlation coefficients, ranging from 0.957 and 0.980, with >95% taxa shared among FL and LB groups (Table 4 and Figure 3B). For the genus and species levels, the Spearman’s correlation coefficients are 0.937 and 0.897, respectively (Table 4), which is presumably due to potential misannotations of shorter contigs in the short-read reference assembly at lower taxonomic units. For the long-read assembly with much greater contig completeness, abundance correlation coefficients remain remarkably high even at the genus (*ρ* = 0.988) and the species levels (*ρ* = 0.979; Table 4), with >99% of FL taxa also identified in LB samples, indicating excellent consistency in taxonomic abundance between the two fecal sample collection approaches.

### The number, alpha diversity, and beta diversity of microbial genes are similar between the LB and FL groups

**Figure 4:**
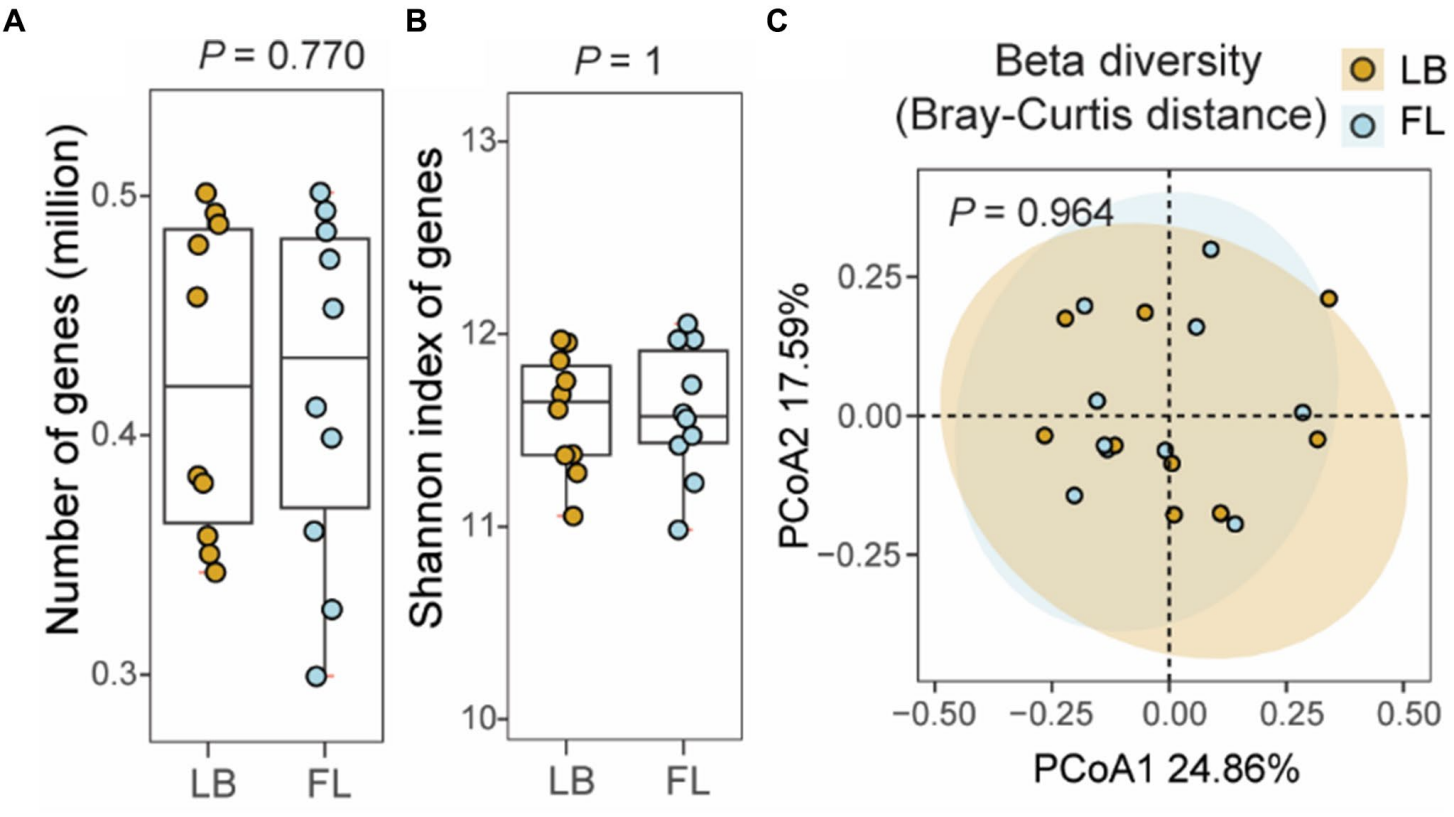
Number of non-redundant microbial genes and gene level diversity in the feline fecal microbiome from samples collected by fecal loop (FL) and litter box (LB) approaches. **(A)** Boxplot of the number of observed genes in the LB (brown) and FL (blue) groups. **(B)** Boxplot of Shannon index of genes identified in the LB (brown) and FL (blue) groups. **(C)** PCoA plot of beta diversity based on Bray-Curtis distance of the genes identified in the LB (brown) and FL (blue) groups.

A total of 860,169 unique microbial genes were identified in the 20 metagenomes. Among these, 10 metagenomes from the LB group contained 796,138 nonredundant genes, while 10 metagenomes from the FL group contained 797,990 nonredundant genes (Figure 4A). Statistical analysis revealed no significant difference in the number of observed genes between fecal samples obtained from the fecal loop and litter box approaches (*p* = 0.770, Wilcoxon signed-rank test; Figure 4A). Additionally, the alpha diversity, as assessed by the Shannon index of observed genes, did not exhibit any significant difference between the two groups (*p* = 1, Wilcoxon signed-rank test; Figure 4B). Furthermore, the PCoA plot based on the Bray-Curtis distance matrix did not reveal any significant dissimilarities between the LB and FL groups, as indicated by the overlapping 95% confidence interval ellipses (*p* = 0.964, PERMANOVA test; Figure 4C). No significant differences were observed in the number, alpha diversity, and beta diversity of the microbial genes identified in the LB and FL groups when long-read assembled feline gut microbiome reference contigs were used as the references (Supplementary Figure S4).

## Discussion

Fecal sample collection plays a crucial role in veterinary medicine for routinely diagnosing various health conditions, including parasitism (Verocai et al., 2020), enteropathogenic bacteria (Marks et al., 2011) and viruses (Sykes, 2014) in research for studying the gut microbiome. Establishing a gold standard for fecal sample collection is crucial for acquiring accurate, reliable, and reproducible microbiome data in a feasible manner. Such a standard safeguards the validity and consistency of microbiome research, facilitating the smooth transition of discoveries into clinical and therapeutic practices. Studies to optimize fecal sample collection techniques were mainly performed for humans, with no specific emphasis on investigating methods tailored for cats. Typically, there are two common methods of collecting feline fecal samples: from the litter box or from the rectum using a fecal loop. Each method possesses its own unique advantages and disadvantages. The fecal loop method is generally considered a more accurate approach for faithfully representing the gut microbiome, as it minimizes the risks of potential cross-contamination and exposure to the environment. However, inserting a fecal loop into the cat rectum requires experienced veterinary professionals to administer sedation, which may not be practical for all situations, particularly in cases where the cat is uncooperative, aggressive, or unable to tolerate sedation due to health concerns. In contrast, fecal samples collected from the litterbox are noninvasive, but more susceptible to environmental contamination, and the duration after defecation may cause bacterial growth to shift the microbiome composition (Vandeputte et al., 2016). Our aim was to conduct a thorough comparison of their impact on microbiome studies to assess whether the two collection methods could be interchangeable under certain circumstances. In this study, we demonstrated that there was no significant difference in the microbial profiles of fecal samples collected from the litter box compared to those collected using a fecal loop. No significant changes were observed in terms of alpha-diversity, beta-diversity, the number of taxa identified at each taxonomic level, and the relative abundances of taxonomic units. Collectively, these findings suggest that the microbiome composition of fecal samples collected using a fecal loop is the same as those collected directly from the litterbox within 6 h post-defecation. This indicates that collecting fecal samples directly from a clean litterbox in a timely manner can be considered a reliable method for feline microbiome studies.

The fecal loop collection approach resulted in a significantly lower DNA yield than the litterbox approach. Due to the uncertainty regarding whether sufficient feces can be collected from the colon in a single trial, the fecal loop method may cause missing data in the research or require multiple collections at different time points, which are not ideal for the experimental design. Consequently, the DNA yield was lower from fecal specimens collected using a fecal loop in this study. If consistent microbial DNA yield is a concern, the litter box approach will guarantee a superior DNA yield compared to the fecal loop approach.

Another disadvantage of using the fecal loop is the possibility of introducing host contaminations to the sample. Our results demonstrated that fecal samples collected using a fecal loop exhibited greater variability in the proportion of host contaminations compared to samples collected from the litter box, although this difference did not reach statistical significance. Notably, one of the fecal samples collected using a fecal loop in this study had a host contamination level of 39%, making it difficult to estimate the necessary sequencing data to achieve the desired depth.

However, using a fecal loop to collect fecal samples remains indispensable for veterinary diagnosis. When fresh feces are needed for medical diagnosis, it is more appropriate to collect fresh fecal samples using a fecal loop in a clinical setting with trained personnel. This method enables the direct assessment of a presenting enteric complaint and the localization to the small, large, or mixed bowel based on fecal features (Sherding and Johnson, 2006), which may be challenging when relying on litter box samples exposed to unknown factors.

For citizen science projects or owner-participated research projects, the fecal loop collection approach is likely not feasible due to the requirement for access to sedation. In such cases, the litter box method is amenable to the participants as it only involves regularly monitoring the litter box. It supports the possibility of applying this feline fecal sample collection method in large-scale population microbiome studies when access to a veterinarian and medical facility is not feasible.

One limitation of our study is that we did not investigate the potential impact of extended room temperature exposure on the microbiome of the fecal samples. In our study, we monitored the litter box every 2 to 6 h to detect fecal deposits. The potential impact of prolonged exposure to room temperature on the composition of the microbiome in fecal samples is an area that requires further exploration.

## Data availability statement

The datasets presented in this study can be found in online repositories. The names of the repository/repositories and accession number(s) can be found at: https://www.ncbi.nlm.nih.gov/, PRJNA1032714.

## Ethics statement

The animal study was approved by Auburn University Institutional Animal Care and Use Committee. The study was conducted in accordance with the local legislation and institutional requirements.

## Author contributions

XM: Data curation, Formal analysis, Investigation, Methodology, Validation, Visualization, Writing – original draft, Writing – review & editing. EB: Investigation, Methodology, Writing – original draft, Writing – review & editing. CL: Conceptualization, Funding acquisition, Resources, Supervision, Writing – review & editing. DD: Conceptualization, Funding acquisition, Resources, Supervision, Writing – review & editing. EC: Conceptualization, Funding acquisition, Resources, Supervision, Writing – review & editing. DM: Conceptualization, Funding acquisition, Resources, Supervision, Writing – review & editing. EG: Conceptualization, Funding acquisition, Investigation, Methodology, Resources, Supervision, Writing – original draft, Writing – review & editing. XW: Conceptualization, Data curation, Formal analysis, Funding acquisition, Investigation, Methodology, Project administration, Resources, Supervision, Writing – original draft, Writing – review & editing.
