# Supplementary material for: Evaluation of fecal sample collection methods for feline gut microbiome profiling: fecal loop vs. litter box
Source: Front Microbiol. 2024 May 10;15:1337917. doi: 10.3389/fmicb.2024.1337917 (PMC11127567; doi:10.3389/fmicb.2024.1337917)

Figure S1. Rarefaction analyses to assess species and gene richness from the results of sampling.

(**A**) Rarefaction curve based on bacterial gene profiles of 20 samples.
(**B**) Rarefaction curve based on taxonomy profiles at the species level of 20 samples.

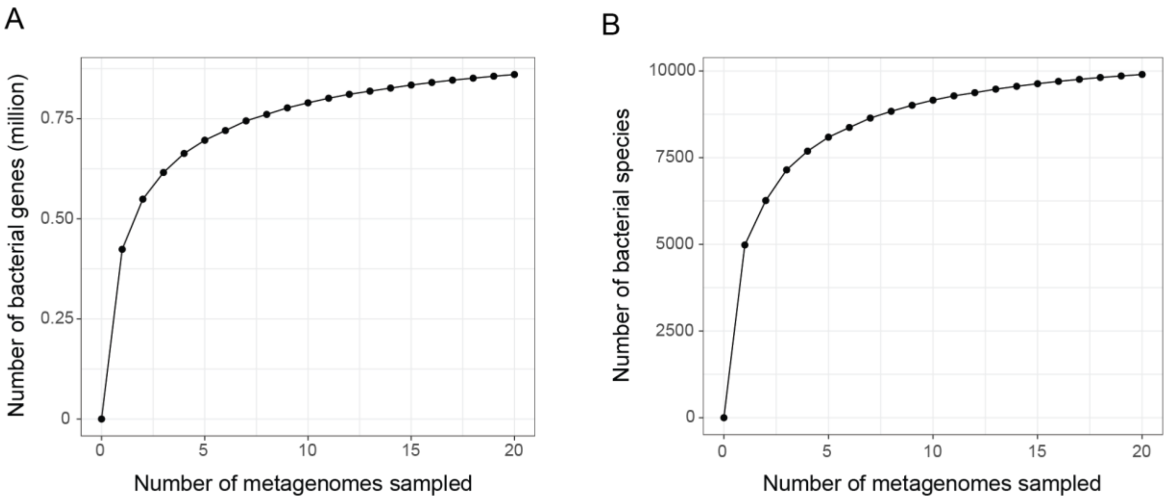

Figure S2. Microbial diversity analyses using long-read reference assembly at different taxonomic levels from fecal samples collected by fecal loop (FL) and litter box (LB) approaches. Boxplots of non-redundant microbial taxa and alpha diversity (Shannon index) for each sample and principal coordinates analysis (PCoA) plot of beta diversity (Bray-Curtis dissimilarity) for microbial profiles from the LB (**brown**) and FL (**blue**) groups at (**A**) phylum, (**B)** class, (**C**) order, (**D**) family, (**E**) genus, and (**F**) species levels. No significant difference was observed in the number of microbial taxa between the LB (15.8 taxa [14.8-16.8 95% CI]) and FL groups (15.6 [14.8-16.4 95% CI]) at the phylum (P = 0.824, Wilcoxon signed-rank test), class (LB: 23.0 [22.4-23.6 95% CI], FL: 22.9 [22.4-23.4 95% CI], P = 0.850), order (LB: 40.6 [38.7-42.5 95% CI], FL: 40.2 [38.4-42.0 95% CI], P = 0.586), family (LB: 69.3 [65.9-72.7 95% CI], FL: 69.5 [66.1-72.9 95% CI], P = 1.000), genus (LB: 228.1 [218.9-237.3 95% CI], FL: 226.0 [217.9-234.1 95% CI], P = 0.210), or species levels (LB: 770.1 [748.1-792.1 95% CI], FL: 764.9 [742.2-787.6 95% CI], P = 0.359). For alpha-diversity, no significant differences were detected at the phylum (LB: 1.06 [1.00-1.12 95% CI], FL: 1.11 [1.01-1.20 95% CI]; P = 0.106), class (LB: 1.66 [1.55-1.76 95% CI], FL: 1.71 [1.62-1.80 95% CI]; P = 0.432), order (LB: 1.74 [1.63-1.85 95% CI], FL: 1.79 [1.70-1.88 95% CI]; P = 0.557), family (LB: 2.25 [2.11-2.40 95% CI], FL: 2.29 [2.17-2.42 95% CI]; P = 0.846), genus (LB: 2.52 [2.35-2.70 95% CI], FL: 2.56 [2.41-2.71 95% CI]; P = 0.922), or species levels (LB: 3.17 [2.98-3.37 95% CI], FL: 3.14 [2.94-3.34 95% CI]; P = 0.625). Similarly, no significant changes were detected in beta-diversity analysis either (P > 0.751 for all taxonomic levels, PERMANOVA test).

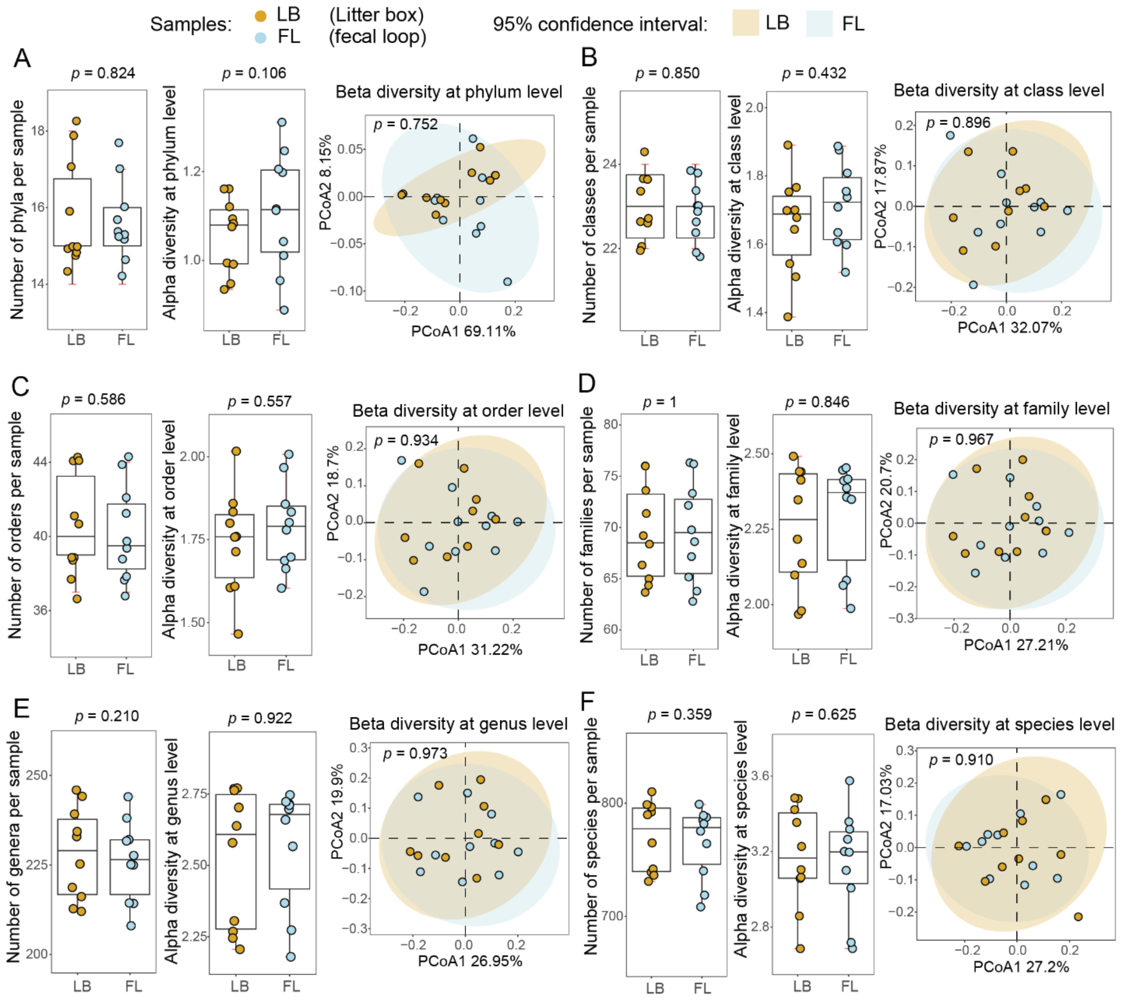

Figure S3. Relative abundance of major phyla and microbiome abundance correlation at class and order levels in the feline microbiome from samples collected by fecal loop (FL) and litter box (LB) approaches from the alignment results against long-read reference assembly.

(**A**) Boxplots of major phyla in LB (**brown**) and FL (**blue**) groups. These five predominant phyla collectively represented more than 96% of all phyla observed in both the LB and FL groups (97.0% [96.2%-97.8% 95% CI] vs 96.8% [96.0%-96.7% 95% CI]; $P = 0.922$). (**B**) Correlation plots of microbes with high abundance (> 0.1%) at class and order levels.

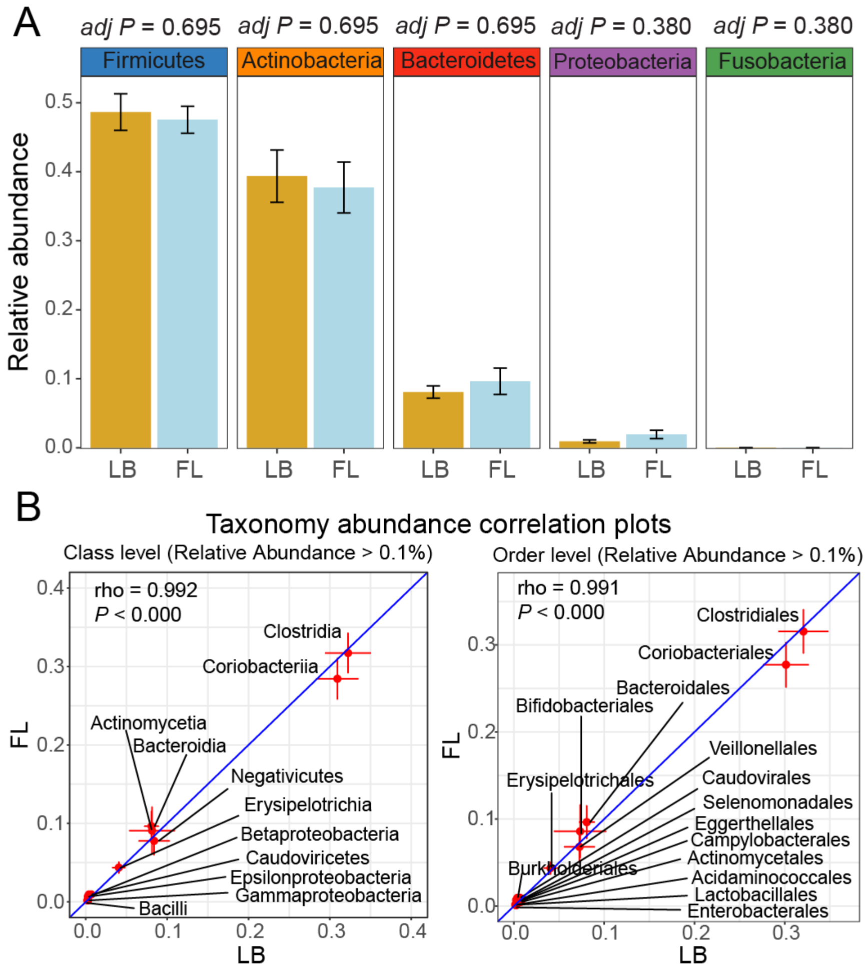

Figure S4. Number of non-redundant microbial genes and gene level diversity in the feline fecal microbiome from samples collected by fecal loop (FL) and litter box (LB) approaches from the alignment results against long-read reference assembly.
(**A**) Boxplot of the number of observed genes in the LB (**brown**) and FL (**blue**) groups. A total of 693,003 unique microbial genes were annotated across the 20 metagenomes. Among these, ten metagenomes from the LB group contained 678,212 nonredundant genes, while ten metagenomes from the FL group contained 678,524 nonredundant genes. Statistical analysis revealed no significant difference in the number of observed genes between fecal samples obtained from the fecal loop and litter box approaches (*P* = 0.770, Wilcoxon signed-rank test). (**B**) Boxplot of Shannon index of genes identified in the LB (**brown**) and FL (**blue**) groups. There was no significant difference in alpha diversity between the two groups (*P* = 0.432, Wilcoxon signed-rank test). (**C**) PCoA plot of beta diversity based on Bray-Curtis distance of the genes identified in the LB (**brown**) and FL (**blue**) groups. There was no significant dissimilarities between the LB and FL groups, as indicated by the overlapping 95% confidence interval ellipses (*P* = 0.961, PERMANOVA test).

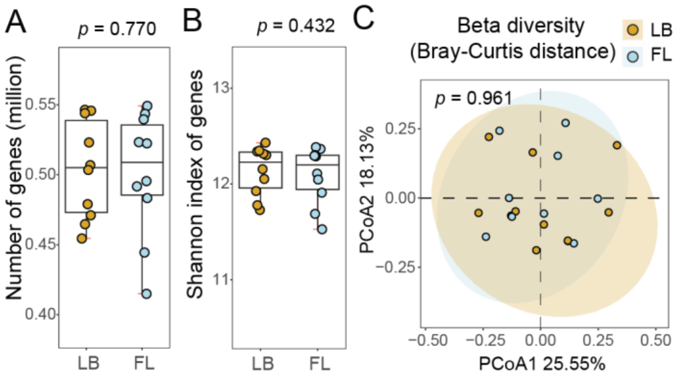

Supplement: Supplementary file 1 [file Data_Sheet_1.pdf]
